# Innovative Strategy on Hydrogen Evolution Reaction Utilizing Activated Liquid Water

**DOI:** 10.1038/srep16263

**Published:** 2015-11-06

**Authors:** Bing-Joe Hwang, Hsiao-Chien Chen, Fu-Der Mai, Hui-Yen Tsai, Chih-Ping Yang, John Rick, Yu-Chuan Liu

**Affiliations:** 1 Department of Chemical Engineering, National Taiwan University of Science and Technology, No. 43, Sec. 4, Keelung Rd., Taipei 10607, Taiwan; 2 Department of Biochemistry and Molecular Cell Biology, School of Medicine, College of Medicine, Taipei Medical University No. 250, Wuxing St., Taipei 11031, Taiwan; 3 Biomedical Mass Imaging Research Center, Taipei Medical University, No. 250, Wuxing St., Taipei 11031, Taiwan; 4 Graduate Institute of Medical Science, College of Medicine, Taipei Medical University, No. 250, Wuxing St., Taipei 11031, Taiwan

## Abstract

Splitting water for hydrogen production using light, or electrical energy, is the most developed ‘green technique’. For increasing efficiency in hydrogen production, currently, the most exciting and thriving strategies are focused on efficient and inexpensive catalysts. Here, we report an innovative idea for efficient hydrogen evolution reaction (HER) utilizing plasmon-activated liquid water with reduced hydrogen-bonded structure by hot electron transfer. This strategy is effective for all HERs in acidic, basic and neutral systems, photocatalytic system with a g-C_3_N_4_ (graphite carbon nitride) electrode, as well as in an inert system with an ITO (indium tin oxide) electrode. Compared to deionized water, the efficiency of HER increases by 48% based on activated water *ex situ* on a Pt electrode. Increase in energy efficiency from activated water is 18% at a specific current yield of −20 mA *in situ* on a nanoscale-granulated Au electrode. Moreover, the onset potential of −0.023 V vs RHE was very close to the thermodynamic potential of the HER (0 V). The measured current density at the corresponding overpotential for HER in an acidic system was higher than any data previously reported in the literature. This approach establishes a new vista in clean green energy production.

Due to growing environmental pollution, renewable fuels, such as hydrogen, are being considered as clean energy sources. Splitting water for hydrogen production using light, or electrical energy, is the most developed ‘green technique’. For increasing efficiency in hydrogen production, currently, the most exciting and thriving strategies are focused on efficient and inexpensive catalysts^1^,^2^,^3^,^4^,^5^,^6^. The electrochemical decomposition of water is possible with visible light of an appropriate energy, while for photochemical splitting a semiconductor-based material is popularly employed^7^,^8^. Importantly, the successes of these technologies for energy conversion in hydrogen production relies on the development of efficient and earth-abundant catalysts^3^,^9^. Other methodologies have been also developed e.g. the construction of a monolithic photovoltaic-photoelectrochemical device^10^, with attempts to decouple hydrogen and oxygen evolution using an electron-coupled-proton buffer^11^. Water has a tetrahedral structure with two O-H bonds that enable it to form a flexible dynamic hydrogen-bonded network, which has been successfully examined using Raman spectroscopy^12^,^13^,^14^,^15^. The facts that increasing the electrolysis temperature can lower the electrolysis voltage for electrolysis^16^ and that water has a more disordered structure with weaker hydrogen bonds at evaluated temperatures^12^ have inspired us to utilize prepared small water cluster (SWC) at room temperature for efficient hydrogen evolution. Based on this strategy, Au nanoparticles (NPs) with well-defined localized surface plasmon resonance (LSPR), are often employed in studies focused on surface-enhanced Raman scattering (SERS)^17^ and in a clinical setting for the photothermal ablation of tumors^18^. In our previous report^19^, they were used to achieve the hot electron transfer needed to break the hydrogen bonds of water. The weak/reduced interaction energy within water molecules provides the potential application in the development of efficient catalyst-free hydrogen production *via* lowering the onset potential. In this work, the prepared pure water with SWC is innovatively utilized for efficient hydrogen evolution reaction (HER). The effect of *in situ* preparation of SWC on the correspondingly increased efficiency in HER is also demonstrated.

## Results and Discussion

### Preparation and characterization of degree of reduced hydrogen bonded water (RHBW)

**Figure 1:**
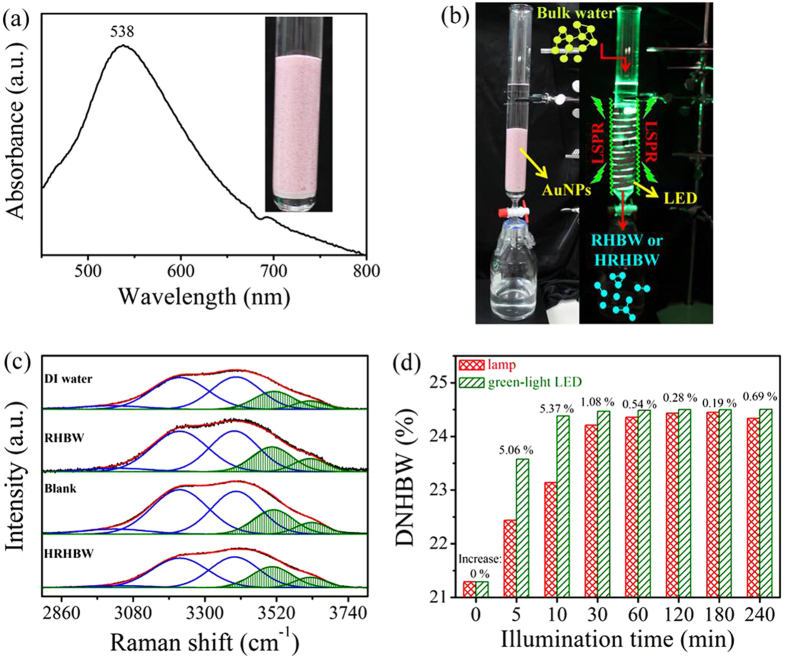
Process of preparing highly reduced hydrogen-bonded water (HRHBW) under resonant illumination on Au NPs. The HRHBW was characterized by Raman spectra. (**a**) Absorption spectrum of the supported Au NPs. (**b**) Schematic setup for the preparation of plasmon-activated liquid water based on the LSPR effect on Au NPs under resonant illumination of an LED (λ_max_ 530 nm). (**c**) Raman spectra of OH stretching of various types of water. (**d**) DNHBW of treated water prepared by using illuminations of fluorescent lamps and green-light LED with time.

Figure 1a indicates that the supported Au NPs in water had a distinct surface plasmon absorption band centered at 538 nm and a broader band extending over the entire visible light region. This LSPR of Au NPs suggests that the effect of hot electron transfer to break hydrogen bonds of bulk water can be achieved under illumination with full-wavelength visible light (to produce RHBW based on fluorescent lamp) and can be further enhanced using light at an optimized wavelength (to produce highly reduced hydrogen bonded water, HRHBW, based on green light-emitting diode). Unless otherwise noted, the blank water was prepared under illumination on deionized (DI) with an indoor fluorescent lamp. In preparation of the blank water, Au NPs were absent. The process of treating water with Au NPs under illumination with a green light-emitting diode (LED, λ_max_ 530 nm) is shown in Fig. 1b.

Figure 1c shows OH-stretching Raman spectra observed with various pure water samples. A blank was obtained using similar preparation conditions to those used to prepare the RHBW, but without illumination. These Raman spectra were further deconvoluted into five Gaussian sub-bands based on methods in the literature^15^,^17^,^18^. Deconvolution of the five-Gaussian components with center wavenumbers of 3018, 3223, 3393, 3506, and 3624 cm^−1^ was adopted for all samples. Moreover, the full widths at the half maximum (FWHMs) of individual components in the five-Gaussian fit were equal for all samples. The respective values were 234, 201, 176, 154, and 112 cm^−1^ for bands at 3018, 3223, 3393, 3506, and 3624 cm^−1^. Bands on the low- and high-frequency sides are respectively related to strongly and weakly hydrogen-bonded OH features. In this work, the three components on the low-frequency side were assigned to hydrogen-bonded water, while the remaining two high-frequency-side components were assigned to non-hydrogen-bonded water. The degree of non-hydrogen-bonded water (DNHBW) was defined as the ratio of the area of the non-hydrogen-bonded OH stretching bands to the area of the total stretching bands. As shown in Table S1, respective values of the DNHBW for DI water, RHBW, blank water, and HRHBW were 21.43 ± 0.05% (21.48% for DI water stored for 4 weeks), 24.32 ± 0.08%, 21.52 ± 0.17%, and 26.33 ± 0.11% (*n* = 3). The smaller difference between values for fresh and stored DI water suggests that 21.43% is a reliable reference value for bulk water used in the DNHBW. Similar values of 21.43% and 21.52% for the DI and blank waters indicate that HRHBW cannot be obtained in the absence of LSPR effect from light-illuminated Au NPs. Encouragingly, the DNHBW significantly increased from 21.43% to 24.32% by utilizing the LSPR effect from the supported Au NPs. This was an increase of 13% for the DNHBW, which could be enhanced to 23% by illumination with a green LED, suggesting that this light is more effective for decreasing hydrogen-bond structures. This effect was also observed in static preparations of treated water based on a fluorescent lamp and a green LED (Fig. 1d and Supporting Information, SI). Moreover, water treatments confirmed the LSPR effect with respect to the corresponding RHBW, with different DNHBWs (Tables S1 and SI). Values of the DNHBW listed in Table S1 range 21% ~ 30%. The energy efficiency, η, when preparing HRHBW was estimated from the ratio of the energy required to break hydrogen bonds in bulk water to that provided from the light energy of the LED, as defined here:


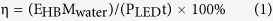


where the energy of hydrogen bonds, E_HB_, of 20 kJ mol^−1^ was used. To obtain 75 g (or 75 cm^3^, using a density of 1 g cm^−3^) of HRHBW, the mole of bulk water, M_water_, in which hydrogen bonds were broken, was calculated from the mole (4.2 mol) multiplied by the difference in DNHBW of DI water (21.46%) and HRHBW (26.23%). The power of the LED used, P_LED_, was 16 W, and the illumination time, t, for 75 cm^3^ of DI water passing through the glass tube was ca. 1500 s. Therefore, the energy efficiency for preparing HRHBW was approximately 4.0 kJ/24 kJ = 17% if energy losses from scattering of the LED and penetrating glass tube of light were ignored.

Samples of DI water, ceramic particle-treated (CPT) water, and Au-coated CPT water were prepared at a temperature of 50 °C and compared to DI water, blank water, and RHBW, which had been prepared at room temperature. The measured Raman spectra of OH stretching of these samples (prepared at 50 °C) at room temperature after cooling in ambient laboratory air are shown in Figure S1. Corresponding values of DNHBW were 21.37%, 21.36%, and 21.35%, respectively, for DI water, CPT water, and Au-coated CPT water. Comparing values of DNHBW of 21.46%, 21.24%, and 26.23% for DI water, CPT water, and HRHBW (prepared at room temperature), respectively, indicates that the amount of joules used for heating contributed less to the successful preparation of HRHBW.

### The effect of electroactive species in (H)RHBW

**Figure 2:**
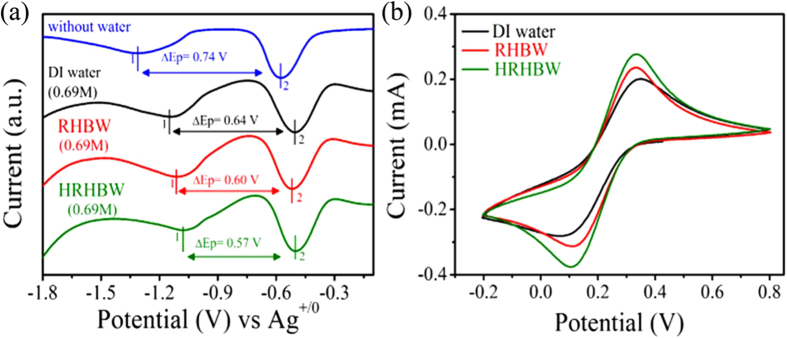
Chemical behaviors of BQ and K_3_Fe(CN)_6_ in the different waters. (**a**) Voltammetric data record for BQ in CH_3_CN containing 0.2 M *n*-Bu_4_NPF_6_ as the supporting electrolyte and different waters with 0.69 M H_2_O at room temperature. (**b**) Voltammetric data recorded in various solutions with 50 mM K_3_Fe(CN)_6_ with a 3-mm-diameter planar Pt electrode.

As recently shown^20^, the electrochemical properties of phenols and quinones in organic solvents are strongly influenced by hydrogen bonds. As shown in Fig. 2a, the first (E_p_^red(1)^) and second (E_p_^red(2)^) reduction peaks of benzoquinone (BQ) shifted toward less-negative potentials, while the potential difference (ΔE_p_) between them decreased when DI water was added to CH_3_CN to form hydrogen-bonded species. These phenomena were more significant with treated water, especially with HRHBW prepared under LED illumination. For example, with H_2_O (0.69 M), decreases in ΔE_p_ were enhanced by 6.3% and 11% (compared to DI water) respectively using RHBW and HRHBW (See Table S2 for reduction peak shifts). This reveals that there was more ‘free water’ in treated water with weaker hydrogen bonding, which could strongly interact with BQ by hydrogen bonding. The evidence of more ‘free water’ was further demonstrated by mixing with ethyl alcohol. DI water and HRHBW both at 10 wt% were individually dissolved in two samples of ethyl alcohol. The measured water contents using volumetric Karl Fischer titration (Metrohm 870 KF Titrino plus) were 10.87 and 10.34 wt% for DI water and HRHBW, respectively^21^. Because the HRHBW with weaker hydrogen bonding could form more hydrogen bonds with ethyl alcohol, the measured content of available water decreased by 4.8%, compared to DI water. Moreover, the novel property of HRHBW was examined by calculating the diffusion coefficient of K_3_Fe(CN)_6_ in water from cyclic voltammetric data through the Randles-Sevcik equation^22^:


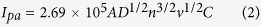


where A is the area of the electrode (0.07 cm^2^ for the Pt electrode used), n is the number of electrons participating in the reaction and was equal to 1, and C is the concentration of the probe molecule in the solution. The calculated diffusion coefficient increased from 6.5 × 10^−6^ to 8.4 × 10^−6^ cm s^−1^ for an increase of 29% when using RHBW instead of conventional DI water (Fig. 2b). Moreover, the diffusion coefficient increased 85% when using HRHBW (12 × 10^−6^ cm s^−1^). In addition, the reduced ΔV (the potential difference between anodic and cathodic peaks in a couple) observed for treated water suggests that the oxidation-reduction reaction of K_3_Fe(CN)_6_ in treated water was more easily reversible.

Also, the chemical activity of steam produced from HRHBW compared to that from DI water was examined with respect to the room-temperature reduction of an Au-containing complex to Au NPs in the presence of Ch. The results suggested that steam produced from HRHBW acts as a weak reducing agent (Figure S2, S3, SI). This discovery opens a new green pathway in chemical reduction.

### Effective hydrogen evolution based on (H)RHBW

**Figure 3:**
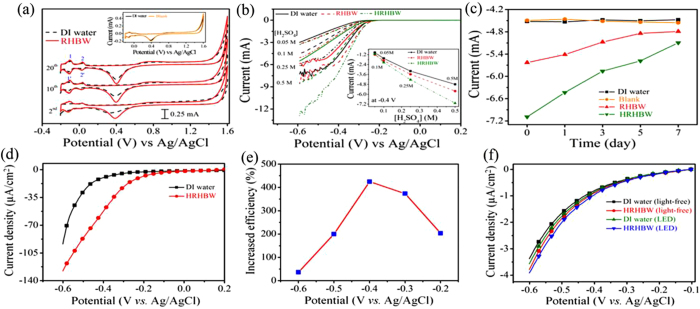
Voltammetric data for hydrogen evolution in various types of water with H_2_SO_4_ as the supporting electrolyte and a planar Pt electrode. (**a**) Cyclic voltammograms of the second, 10^th^ and 20^th^ scans in 0.5 M H_2_SO_4_ at a scan rate of 0.5 V s^−1^; the insert shows the 10^th^ scans for experiments performed on DI water and the blank (light-free) for comparison. (**b**) Linear sweep voltammograms (LSVs) in different concentrations of H_2_SO_4_ at a scan rate of 0.05 V s^−1^; the insert shows the hydrogen evolution currents at −0.4 V vs Ag/AgCl in various waters with different concentrations of H_2_SO_4_. (**c**) Currents of hydrogen evolution at −0.4 V vs Ag/AgCl in various types of water, after its preparation for 0, 1, 3, 5, and 7 days (in 0.5 M H_2_SO_4_ at a scan rate of 0.05 V s^−1^). Hydrogen evolution reaction performed at an inert-catalytic electrode (ITO) and photocatalytic (g-C_3_N_4_/ITO) electrodes. (**d**) LSVs for hydrogen evolution in DI water and HRHBW containing 0.5 M H_2_SO_4_ based on the ITO electrode. (**e**) The increased efficiency of HER based on HRHBW with the applied potential shown in Fig. 3d. (f) LSV for hydrogen evolution reactions performed in DI water and HRHBW containing 0.5 M Na_2_SO_4_ based on g-C_3_N_4_/ITO electrode with and without illumination of white-light LED.

The weak hydrogen-bonded water encouraged us to evaluate it for efficient hydrogen evolution reaction, reasoning that less energy would be needed to electrolyze HRHBW. As shown in Fig. 3a regarding CV in the second scan, the two pairs of near-reversible peaks, associated with hydrogen desorption-adsorption at <0.1 V, were not well-defined in DI water, but were well-defined in RHBW^23^,^24^. Similar CV data recorded for the DI and blank waters again suggested that lacking illumination would not change the water’s property (Figure S4, S5, SI). After the 10^th^ scan, the two pairs of peaks were well-defined in experiments performed with both DI water and RHBW; however, the areas associated with hydrogen desorption-adsorption in RHBW were significantly larger. After the 20^th^ scan, the decreases in ΔV from 0.019 to 0.009 V for hydrogen desorption-adsorption (1 and 1′) and from 0.024 to 0.005 V for hydrogen desorption-adsorption during underpotential deposition (2 and 2′) observed in RHBW suggested that the hydrogen desorption-adsorption reaction in RHBW was more easily reversible^23^,^24^. The anodic vertex showed that the corresponding onset potential of oxygen evolution was lower in RHBW, especially in the second scan.

As shown in Fig. 3b regarding the linear sweep voltammogram (LSV) performed on DI water, RHBW, and HRHBW, the onset potential of the cathodic HER was more positive in RHBW and especially so in HRHBW, suggesting that the required energy for the HER could be reduced through the weakening of hydrogen bonds. HER currents were recorded and compared at −0.4 V (see insert of Fig. 3b), to avoid interference from the produced hydrogen bubbles. As expected, absolute values of the currents increased with increases in electrolyte concentrations. Encouragingly, the HER was significantly promoted in treated water. Compared to DI water, the efficiency of hydrogen evolution respectively increased by ca. 17% and 48% using RHBW and HRHBW [in 0.5 M H_2_SO_4_ at −0.4 V (Table S3)]. Moreover, the increased efficiency of metastable treated water was dependent on the storage time (Fig. 3c). In addition, the calculated Faraday efficiencies in hydrogen productions are 84.0 ± 5.4% and 86.2 ± 3.5% for systems of DI water and HRHBW (See SI).

The Pt electrode is recognized as an excellent catalyst for electrolyzing water. The above result demonstrates that HRHBW possesses efficient hydrogen evolution reaction compared to DI water based on the Pt-electrode system. To exclude the effect of Pt during electrolysis, an indium tin oxide (ITO) electrode was substituted for the Pt electrode. As shown in Fig. 3d, compared to the Pt electrode, the current at −0.6 V was much smaller, indicating that the ITO electrode was not favorable for electrolyzing water. In addition, the increased efficiencies of HER at potentials of –0.2, –0.3, –0.4, –0.5 and –0.6 V are 204%, 374%, 424%, 200% and 36.2%, respectively (Fig. 3e). The continuously increased efficiency in cathodic scan from –0.2 to –0.4 V is ascribed to the lower required onset potential in HER utilizing HRHBW instead of DI water. At lower cathodic potential, the difference in efficiencies of HER is predominantly decided by the difference of interactions within water molecules. However, this superiority is reduced as the cathodic potential increases further from –0.4 to –0.6 V. It indicates that more efficient water splitting accompanied by overcoming the difference of interactions within water molecules between HRHBW and DI water is occurred as the applied cathodic overpotential is high enough. In comparison, discussion regarding overpotential is based on the same reference electrode in this work. These results reveal that high efficient HER can be also performed in the HRHBW-based system even using inert ITO electrode.

Moreover, famous HER catalyst of g-C_3_N_4_ was deposited on ITO electrode to evaluate the effect from utilizing HRHBW on the increased efficiency of HER in 0.5 M Na_2_SO_4_. g-C_3_N_4_ has drawn consider considerable attention in recent years for its catalytic activity in visible-light photocatalytic water splitting^25^,^26^. It is a visible-light-active polymeric semiconductor with a band gap of ∼2.7 eV, corresponding to an optical wavelength of ∼460 nm^27^. This appropriate band structure makes it available for both efficient water reduction and oxidation. As shown in Fig. 3f, the recorded current density on the g-C_3_N_4_/ITO electrode in HRHBW system is higher than that in DI water system in the cathodic scan from 0 to –0.6 V. In light free condition, the current density is −3.79 μA cm^−2^ at –0.6 V for HRHBW system. It is an increase of 12.1% in HER efficiency compared to the DI water system (−3.38 μA cm^−2^). In addition, the current densities obtained in light free condition increase 5.6% and 3.4% for DI water and HRHBW, respectively, under illumination of white-light LED. Compared to the DI water system, the lower increased current density obtained in HRHBW system can be attributed to the lower effect of charge transfer from photocatalyst of g-C_3_N_4_ to water under illumination. In preparation of HRHBW this similar charge transfer had occurred on treated DI water from hot electron transfer^19^. Thus, charge transfer from g-C_3_N_4_ to HRHBW contributes less to HER in electrolytic water splitting. Under illumination of white-light LED, the current density is −3.92 μA cm^−2^ at –0.6 V for HRHBW system. It is an increase of 9.8% in HER efficiency compared to the DI water system (−3.57 μA cm^−2^). Conclusively, these results suggest that the utilizing of HRHBW in the improvement of HER is available for all types of electrodes, including active (Pt), photocatalytic (g-C_3_N_4_) and inert (ITO) ones. Further inductively coupled plasma-mass spectrometric (ICP-MS) analysis indicated that the concentration of slightly dissolved Au metals in the RHBW was ca. 0.62 ppb (ca. 0.65 ppb for HRHBW). The dissolved Au ions may be deposited on the Pt electrode, and this may contribute to the increased current in HER. This influence of slightly dissolved Au ions in the RHBW on the correspondingly efficient HER is excluded, as shown in Figure S7 (Similar experiments as performed in Fig. 3). After the first HER on polished Pt electrode based on DI water, the used Pt electrode was just rinsed with DI water without further polishing treatment in sequent experiments based on DI water, HRHBW, DI water and HRHBW in sequence. The HERs in the first and the third experiments based on DI water are similar. The HERs in the second and the fourth experiments based on HRHBW are similar. Therefore, the influence of slightly dissolved Au ions on the effective HER in HRHBW is excluded.

**Figure 4:**
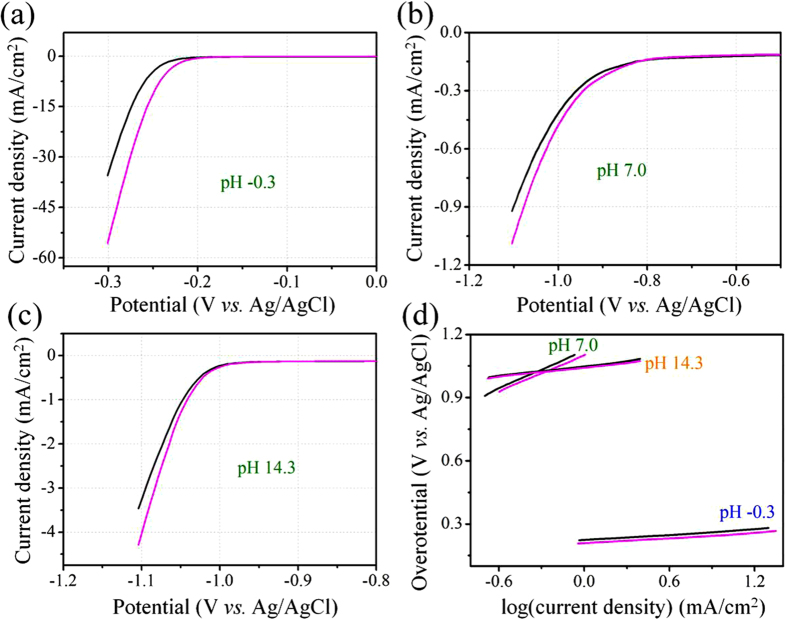
Hydrogen evolution reactions performed at inert-catalytic electrode (ITO) in electrolyte with various pH values. LSV curves recorded on Pt electrode in (**a**) 1 M H_2_SO_4_ (pH −0.3), (**b**) 0.2 M KCl (pH 7), and (**c**) 2 M KOH (pH 14.3). (**d**) The corresponding Tafel plots at pH values of 0.3, 7.0 and 14.3. Black line: electrolyte solution based on DI water. Pink line: electrolyte solution based on HRHBW water.

In addition, the effects of HRHBW with weak hydrogen bonds on the corresponding hydrogen evolution reaction in neutral and alkaline systems were further evaluated. As shown in Fig. 4a, in the HRHBW-based system, the overpotential (at 10 mA cm^−2^) at pH −0.3 was 0.24 V, reduced 0.03 V compared to DI water-based system. Also, in the HRHBW-based system, the overpotential at 1 mA cm^−2^ was 0.21 V, reduced 0.01 V compared to DI water-based system. In addition, it had a smaller Tafel slope (43.0 mV decade^−1^) (Fig. 4d).

Similarly, reduced overpotentials and smaller Tafel slopes of the HRHBW-based systems were also observed at pH 7.0 and 14.3 (Fig. 4b–d, Table S4). It is noteworthy that unlike the acid electrolyte, achieving high efficiency of hydrogen evolution reaction in neutral and alkaline systems is considered a formidable challenge. For instance, the HER in acidic medium is higher by about 2 ~ 3 orders of magnitude than in alkaline medium^23^. Still, compared to DI water-based systems, Tafel slopes of HRHBW-based systems at all pH values decreased by nearly 5%. This clearly shows that interactions within water molecules are one of the main factors in improving the efficiency and will be useful for all pH systems. Also, as discussed in SI, hydrogen bonds within water molecules can be directly broken on the electrochemically roughened Au substrate under fluorescent illumination.

**Figure 5:**
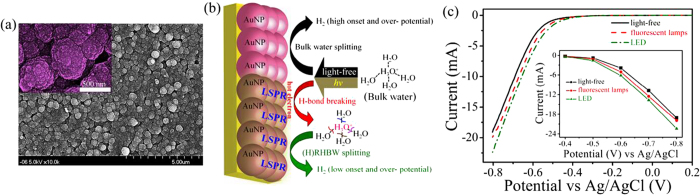
Linear sweep voltammograms of hydrogen evolution in DI water containing 0.5 M H_2_SO_4_ at an electrochemically roughened Au electrode under illumination with different light sources. (**a**) SEM image of the roughened Au electrode (0.283 cm^2^) which was prepared by using electrochemical oxidation-reduction cycles (ORCs). (**b**) The sketch reveals the combination of *in situ* producing and splitting (H)RHBW). (**c**) LSVs of hydrogen evolution in DI water containing 0.5 M H_2_SO_4_ at an electrochemically roughened Au electrode with different illumination sources (lamp and green-light LED).

Therefore, the *in situ* reduction of water’s hydrogen bonds combining the HER was evaluated on the Au NP-deposited Au electrode under illumination. An SEM image shows that abundant Au NPs were deposited on the Au electrode by an electrochemical oxidation-reduction cycle (ORC) method (Fig. 5a). In addition, the mechanism of improving the HER through synchronously forming and electrolyzing HRHBW was proposed (Fig. 5b). It can be observed that the onset potential of the cathodic HER was more positive when the roughened Au electrode was illuminated with a fluorescent lamp, and especially so under green LED illumination (Fig. 5c). As expected, the HER was significantly promoted when the Au electrode was illuminated with a fluorescent lamp (compared to a light-free condition) or with a green LED. Compared to the light-free condition, the efficiency of hydrogen evolution was significantly increased by ca. 31% and 59% based on experiments performed under illuminations of lamps and LED, respectively (Tables S5, SI). This increase was enhanced to 84% based on *in situ* formation of HRHBW using the green LED when DI water was further replaced by HRHBW (Figure S6). Moreover, the increased energy efficiencies from HRHBW compared to bulk water were 18%, 16%, and 14% at specific current yields of −20, −30, and −40 mA, respectively (See SI).

In the recent literature reporting on improvements of the HER efficiency in water splitting, research focused on reducing costs in HER. In water electrolysis, two strategies are generally adopted for improving efficiency: lowering the cathodic overpotential and increasing the specific surface area of the electrode. Although Pt-based electrodes are widely deemed useful as catalysts in water electrolysis, the high cost of this noble metal is a concern. Therefore, scientists have attempted to develop cheaper but novel materials to fabricate efficient electrodes^28^,^29^,^30^,^31^,^32^,^33^. However, on the other hand, investigations regarding interactions within intrinsic water molecules that affect electrolysis efficiency have generally been neglected. Our experimental results indicate that the onset potentials, which are defined as the cathodic currents, rise rapidly as more negative potentials are applied, and on the Pt cathode using DI water-, RHBW-, and HRHBW-based electrolytes were −0.268 (−0.046), −0.253 (−0.031), and −0.245 (−0.023) V vs AgCl (vs RHE). These onset potentials for the RHBW- and HRHBW-based electrolytes were very close to the thermodynamic potential of the HER (i.e., 0 V vs RHE). To the best of our knowledge, the current density at the corresponding overpotential for the HER with HRHBW-based electrolytes is higher than anything reported in the literature for systems of Pt or Au catalysts in acidic electrolytes (Table 1)^31^,^32^,^33^,^34^,^35^,^36^. It should be kept in mind, however, that unlike the reported Pt-modified electrodes with high surface areas in DI water-based electrolytes, even with a smooth Pt electrode as used in this work, the HRHBW-based electrolyte exhibited the highest HER efficiency.

Experimental results show that water molecules with reduced hydrogen binding are easily electrolyzed. These results are consistent with the fact revealed from the density functional theory (DFT), in which the interaction energy of H_3_O^+^–OH^−^ is 46.9 kJ mol^–1^; while this energy is increased by ca. 2.5 times when H_3_O^+^ is associated with an additional four water molecules by hydrogen bonds^37^. In this work, we propose one basic but important relationship between the interaction of water molecules and the HER efficiency on both Pt and Au electrodes. Obviously, lowering the onset potential and overpotential of the HER is feasible by reducing the hydrogen bonds of water molecules. This innovative approach is potentially applicable to currently developed catalysts to further improve their HER efficiencies.

In summary, we have innovatively utilized the LSPR of Au NPs to prepare water with reduced hydrogen bonds. The reduced hydrogen-bonded water possesses many novel properties, including efficient HERs in water splitting. This establishes a new vista in clean green energy production. We believe these new approaches based on the prepared RHBW will lead to a variety of applications, in medicine, biology and chemistry.

## Methods

### Chemicals and Materials

Sodium chloride, tetra-n-butylammonium hexafluorophosphate (n-Bu_4_NPF_6_, 98%), sulfuric acid, and hydroquinone were purchased from Sigma-Aldrich Organics. Acetonitrile was purchased from Tedia. All of the reagents were used as received without further purification. The 40-mesh-screened ceramic particles (molar compositions: 92% SiO_2_, 3.0% Na_2_O, and K_2_O, 2.0% Fe_2_O_3_, 1.5% Al_2_O_3_, 0.5% CaO, 0.5% MgO, and other rare metal oxides) for filtering through deionized (DI) water were purchased from Chyuan-Bang Enterprise, Taiwan. Commercial chitosan (Ch) powder with a degree of deacetylation of 0.82 was purchased from First Chemical Works, Taiwan. All of the solutions were prepared using DI water (18.2 MΩ cm) provided by a Milli-Q system. All of the experiments were performed in an air-conditioned room at ca. 24 °C. The water temperature was ca. 23.5 °C.

### Preparation of Gold Nanoparticles

Au NPs in an aqueous solution were obtained from an Au sheet (with a purity of 0.9999) using electrochemical and thermal reduction methods^38^. Typically, an Au electrode was cycled in a deoxygenated aqueous solution (40 mL) containing 0.1 N NaCl and 1 g L^−1^ chitosan (Ch) from −0.28 to 1.22 V vs Ag/AgCl at 500 mV s^−1^ for 200 scans under light stirring. Durations at the cathodic and anodic vertices were 10 and 5 s, respectively. Immediately, without changing the electrolytes, the solution was heated from room temperature to boiling at a heating rate of 6 °C min^−1^ in air. After cooling, the clear, Au NP-containing solution was separated from the settled Ch. Then the Au NP-containing solution was placed in an ultrasonic bath for 30 min and was further centrifuged at 3600 rpm for 2 min to remove any Ch to prepare pure Au NPs in solution.

### Preparation of Cramic Prticles-supported Au NPs

The rinsed ceramic particles were immersed in a solution containing 30 ppm AuNPs for 1 day. Then the AuNPs-adsorbed ceramic particles were rinsed throughout with DI water, and finally dried in an oven at 100 °C for 1 day. Subsequently, the prepared AuNPs-adsorbed ceramic particles were loaded in a valve-equipped glass tube (I.D.: 30 mm, L: 300 mm). Before treating water the AuNPs-adsorbed ceramic particles in the glass tube were rinsed with DI water for several cycles until the pH value is constant (ca. pH 7.23 and water temp. at ca. 23.5 °C).

### UV-vis spectrum of ceramic particles-supported Au NPs

Ultraviolet-visible absorption measurement for the ceramic particles-supported Au NPs was carried out by using a Perkin-Elmer Lambda 800/900 spectrophotometer. For measuring the spectrum of the solid ceramic particles-supported Au NPs based on a reflection model the spectrophotometer was equipped with a collector of integrating sphere. Wetted ceramic particles were used as the background reference in experiment.

### Preparation of Reduced Hydrogen-bonded Water (RHBW) and Highly RHBW (HRHBW)

DI water (pH 7.23, T = 23.5 °C) was passed through a glass tube filled with AuNPs-adsorbed ceramic particles under illumination. Water treated under illumination with a fluorescent lamp was named RHBW, and that treated under a green light-emitting diode (LED) was named HRHBW. Then, the treated water (pH 7.25, T = 23.3 °C) was collected in glass sample bottles for subsequent tests as soon as possible. To examine the purity of the prepared RHBW, further inductively coupled plasma-mass spectrometric (ICP-MS) analyses indicated that the concentrations of slightly dissolved metals in the RHBW were ca. 0.62 ppb for Au, 43 ppb for Na, 25 ppb for K, 23 ppb for Al, 13 ppb for Mg, 4.5 ppb for Ca, and 0.41 ppb for Fe. Excluding Au, the total equivalent molar concentration of these dissolved metals was equal to ca. 6.9 × 10^−6^ N. The measured value of the dissolved metals for DI water was ca. 2.4 × 10^−7^ N as a reference. Also, the slightly dissolved Au and the total equivalent molar concentration of the other dissolved metals in the blank water were 0.57 ppb and 5.2 × 10^−6^ N, respectively.

### Raman Spectra Recorded on Water and Their Deconvolutions

The prepared water was sealed in a 0.5-mL cell with a glass window. Raman spectra were obtained (Micro Raman spectrometer, Model UniRAM-Raman) with a confocal microscope employing a diode laser operating at 532 nm with an output power of 1 mW on the sample. A 50×, 0.36 NA Olympus objective (with a working distance of 10 mm) was used to focus the laser light on the samples. The laser spot size was ca. 2.5 μm. A thermoelectrically cooled Andor iDus charge-coupled device (CCD) of 1024 × 128 pixels operating at −40 °C was used as the detector with 1-cm^−1^ resolution. All spectra were calibrated with respect to a silicon wafer at 520 cm^−1^. In measurements, a 180° geometry was used to collect the scattered radiation. A 325 notch filter was used to filter the excitation line from the collected light. The acquisition time for each measurement was 1 s. Thirty sequential measurements were collected on each sample.

### Time for preparing reduced hydrogen-bonded water (RHBW) using different light sources

Two samples of DI water (20 mL) were added in sealed glass sample cells (50-mL), which were placed together on a platform of an orbital shaker and operated at 150 rpm. Every cell contained ceramic particle-supported Au NPs (ca. 20 mL) at the bottom for treating the water under illumination with a fluorescent lamp (RHBW) or green LED (HRHBW). The treated water samples were taken at certain times, and their corresponding Raman spectra were measured. Values of the degree of non-hydrogen-bonded water (DNHBW) were calculated as the method and definition mentioned before and shown in the text, respectively.

### Chemical activity of water steam in reduction preparation of Au NPs

First, an Au-containing complex (ca. 250 ppm) was electrochemically prepared in DI water using a method similar to that described in a previous report^39^. Typically, when preparing the Au-containing complex, the Au electrode was cycled in a deoxygenated aqueous solution containing 1 M NaCl from −0.28 V (held for 10 s) to 1.22 V (held for 5 s) at 500 mV s^−1^ for 500 scans. Subsequently, 2 g L^−1^ of Ch was added to the Au complex-containing solution under light stirring for 10 min to prepare a stock solution as a precursor of Au NPs. Then 10-mL stock solutions were dropped onto qualitative filter paper (with pore size of 11 μm and a diameter of 90 mm, lot no. 10809161, Toyo Roshi Kaisha, Japan). The wetted paper was immediately placed on the opening of two glass sample cells (50 mL), which each contained DI water and highly RHBW (HRHBW) of 40 mL, in ambient laboratory air for 3 days. The oxidation states of the Au NPs and Au salts (precursors of Au NPs) were examined using high-resolution x-ray photoelectron spectroscopy (HRXPS). In the measurements, a ULVAC PHI Quantera SXM spectrometer with monochromatized Al K_α_ radiation, at 15 kV and 25 W, and an energy resolution of 0.1 eV was used. To compensate for surface-charging effects, all HRXPS spectra are referenced to the C 1 s neutral carbon peak at 284.8 eV.

### Electrochemical Measurements

The electrochemical behavior of K_3_Fe(CN)_6_ was evaluated in a three-electrode system consisting of a Pt electrode (0.07 cm^2^), a Pt sheet, and Ag/AgCl as the working, counter and reference electrodes, respectively. Measurements were obtained in water with 50 mM K_3_Fe(CN)_6_ at a scan rate of 0.1 V s^−1^.

### Hydrogen Production Measurements

Hydrogen evolution reaction (HER) was measured by linear sweep voltammetry (LSV) in a three-electrode system consisting of a polished Pt electrode (0.07 cm^2^) or ITO electrode (0.28 cm^2^), a Pt sheet, and Ag/AgCl as the working, counter, and reference electrodes, respectively. The corresponding electrochemical measurement was performed in a 50-mL solution with 0.5 M H_2_SO_4_ at a scan rate of 0.05 V s^−1^. Before HER, the aqueous solution had been carefully deoxygenated by bubbling highly purified nitrogen for 30 min.

### Preparation of graphite carbon nitride (g-C_3_N_4_)

Catalyst of graphite carbon nitride (g-C_3_N_4_) was synthesized by thermal treatment of urea (30 g) in an alumina crucible with a cover at 550 °C in a Muffle furnace for 3 h. The obtained powders were rinsed with DI water and ethanol to remove any residual alkaline species and then collected by filtration before drying. 10 μL of g-C_3_N_4_ (0.5 g mL^−1^) was mixed with 10 μL of dimethyl sulfoxide. Then the mixture was dropped onto the indium tin oxide (ITO) glass (1 cm^2^) before heating at 200 °C for 1 h to improve adhesion of catalyst on ITO electrode.

## Additional Information

**How to cite this article**: Hwang, B.-J. *et al.* Innovative Strategy on Hydrogen Evolution Reaction Utilizing Activated Liquid Water. *Sci. Rep.* **5**, 16263; doi: 10.1038/srep16263 (2015).

## Supplementary Material

Supplementary Information

## Figures and Tables

**Table 1:** Comparison of the efficiency of HER based on Pt and Au electrodes in H_2_SO_4_ electrolytes.

Electrode	Current density (*j*, mA cm^−2^)	Overpotential at the corresponding *j*	[H_2_SO_4_]	ref.
Pt/C^c^	10.7	−0.3 V	0.50 M	31
Pt (Au NT water)	22.8	−0.3 V	0.50 M	This work
	80.7	−0.4 V		
Pt (Au NT water-LED)	27.2	−0.3 V	0.50 M	This work
	100.2	−0.4 V		
Pt/Nanoporous Au	45	−0.4 V	0.50 M	32
Pt/4-sulfophenyl/GC	1.77	−0.4 V	0.50 M	33
PW_12_O_40_^3-^/PtNP/ITO	5.3	−0.4 V	0.50 M	34
Pd_60_Pt_40_^b^	64	−0.4 V	0.50 M	35
Ppy_0.2_(Pt)/Pt^c^	1.4	−0.3 V	0.05 M	36
Pt (Au NT water)	3.2	−0.3 V	0.05 M	This work
Pt (Au NT water-LED)	3.7	−0.3 V	0.05 M	This work
Au	43	−0.8 V	0.50 M	32
Nanoporous Au	62	−0.8 V	0.50 M	32
ORC-Au *in situ* (LED)	79.3	−0.8 V	0.50 M	This work

^a^Reference electrode of RHE transfers into Ag/AgCl; E_vs RHE_ = E_vs Ag/AgCl _+ E^0^_Ag/AgCl _+ 0.059 pH (at 298 K).

^b^Reference electrode of NHE transfers into Ag/AgCl; E_vs NHE_ = E_vs Ag/AgCl _+ E^0^_Ag/AgCl _+ 0.059 pH (at 298 K).

^c^Reference electrode of SCE transfers into Ag/AgCl; E_vs RHE_ = E_vs SEC _+ E^0^_SEC _+ 0.059 pH (at 298 K).
