# Supplementary material for: Innovative Strategy on Hydrogen Evolution Reaction Utilizing Activated Liquid Water
Source: Sci Rep. 2015 Nov 6;5:16263. doi: 10.1038/srep16263 (PMC4635376; doi:10.1038/srep16263)
Supplement: Supplementary Information [file srep16263-s1.doc]

Supplementary Information

Innovative Strategy on Hydrogen Evolution Reaction Utilizing Activated Liquid Water

Bing-Joe Hwang ^1^, Hsiao-Chien Chen ^2^, Fu-Der Mai ^2,3^, Hui-Yen Tsai ^2^, Chih-Ping Yang^4^, John Rick ^1^ & Yu-Chuan Liu ^2,3^,*

^1^ Department of Chemical Engineering, National Taiwan University of Science and Technology, No. 43, Sec. 4, Keelung Rd., Taipei 10607, Taiwan ^2^ Department of Biochemistry and Molecular Cell Biology, School of Medicine, College of Medicine, Taipei Medical University No. 250, Wuxing St., Taipei 11031, Taiwan. ^3^ Biomedical Mass Imaging Research Center, Taipei Medical University, No. 250, Wuxing St., Taipei 11031, Taiwan. ^4^ Graduate Institute of Medical Science, College of Medicine, Taipei Medical University, No. 250, Wuxing St., Taipei 11031, Taiwan.

B.-J. Hwang and H.-C. Chen. contributed equally to this work. Correspondence and requests for materials should be addressed to Y.-C. Liu, (E-mail: [liuyc@tmu.edu.tw](mailto:liuyc@tmu.edu.tw)).

**Supplementary Discussion**

1. DNHBWs on differently treated water

As demonstrated in Table S1, ceramic particle-treated (CPT) water was obtained by similar preparation conditions to those used to prepare RHBW, but without Au NPs on the ceramic particles. A super HRHBW Raman spectrum was obtained by irradiating DI water-wetted ceramic-supported Au NPs with laser light at 532 nm. Similar values of 21.46% and 21.24% for DI and CPT waters show that HRHBW cannot be obtained when just using ceramic particles in the absence of the LSPR effect from the Au NPs. The DNHBW of super HRHBW was 30.31%, which was much higher than the value of HRHBW. To consider the effect of temperature, a similar experiment was performed as when preparing the super HRHBW, but DI water was used to wet the Au NP-free ceramic particles. The obtained DNHBW was 21.90%, indicating that temperature only exerted a slight effect on the observed extremely high DNHBW of the super HRHBW. Also, no asymmetric stretching vibrations of isolated water molecules were observed at ca. 3755 cm^-1^, suggesting that water vapor can be excluded from this HRHBW^40^. Ag and Pt NPs instead of Au NPs were also used to prepare RHBW under illumination with a fluorescent lamp. The results were also positive. Values of DNHBW were 24.36% and 24.23% based on Ag and Pt NPs, respectively. Moreover, Au/Ag composite NPs compared to Au NPs contributed a little more to the preparation of RHBW under illumination with the same fluorescent lamp (24.94% DNHBW). It is well known that the catalytic activity of supported Au NPs is higher than that of unsupported Au NPs. In this work, Au NPs were adsorbed onto ceramic particles, in which the main component was SiO_2_. Therefore, Au NPs were deposited on an Au substrate (D: 6 mm) using electrochemical oxidation-reduction cycles (ORCs) to examine the supporting effect on the corresponding DNHBW (500 cycles were used in this experiment). This Au NP-deposited substrate was immersed in 20 mL of DI water in a glass bottle. Then the bottle was placed on a shaking platform under illumination with a fluorescent lamp for 2 h. The corresponding DNHBW was 22.86%, which was higher than the value of DI water. This reveals that Au NPs themselves can break down hydrogen bonds of water under illumination with resonant light to form RHBW. Moreover, as discussed in the text, values of DNHBW for DI water, RHBW, blank water, and HRHBW, which are based on Figure 1c in the text, were 21.46%, 24.24%, 21.50%, and 26.23%, respectively. Moreover, average values of DNHBW for DI water, RHBW, blank water, and HRHBW, which were based on three different batch experiments, were 21.35±0.11%, 24.27±0.15%, 21.52±0.07%, and 26.21±0.07%, respectively. Compared to significant differences between values of the DNHBW for the four water samples, the slight errors in these four individual values of DNHBW are acceptable.

2. Time for preparation of treated water by using different lights

As shown in Figure 1d, the DNHBW significantly increased from 22.44% to 23.57% by utilizing the LSPR effect from the supported Au NPs illuminated with a green LED instead of a fluorescent lamp for 5 min. The DNHBW increased by 5.0%. This increase was gradually reduced after illumination for 120 min when the DNHBW of RHBW reached a maximum value of ca. 24.43%. However, illumination with the green LED for ca. only 10 min was necessary to achieve the same level of this maximum value. This suggests that resonant light from the green LED was more capable.

3. Joule heating contributes less to the successful preparation of HRHBW

Samples of DI water, CPT water, and Au-coated CPT water were prepared at a temperature of 50 °C and compared to the DI water, blank water, and RHBW, which had been prepared at room temperature. The measured Raman spectra of OH stretching of these samples (prepared at 50 °C) at room temperature after cooling in ambient laboratory air are shown in Figure S1. The corresponding values of DNHBW were 21.37%, 21.36%, and 21.35%, respectively, for DI water, CPT water, and Au-coated CPT water. Comparing values of the DNHBW of 21.46%, 21.24%, and 26.23% for DI water, CPT water, and HRHBW (prepared at room temperature), respectively, indicates that joule heating contributed less to the successful preparation of HRHBW.

4. Mechanisms of the formation of HRHBW and persistence in liquid water

As reported in the literature^41-43^, light-induced vapor generation at water-immersed Au NPs was enabled when Au NPs were illuminated with solar energy or resonant light of sufficient intensity. However, the threshold of resonant light intensity was (~10^6^ W m^-2^). In this work, when preparing HRHBW, weakly hydrogen-bonded DI water (pH 7.23, T = 23.5 °C) was passed through a glass tube (with a diameter of 3 cm) filled with Au NP-adsorbed ceramic particles (with a height of 15 cm) under illumination. Then HRHBW (pH 7.25, T = 23.3 °C) was collected as soon as possible in glass sample bottles for subsequent tests. When illuminated with a full visible wavelength fluorescent lamp (RHBW), or alternatively a green LED (wavelength maxima centered at 530 nm, HRHBW), the light power density at resonant 538 nm was ca. 10^-3^ or 10^-2^ W m^-2^, respectively, which is far lower than that of the required threshold for light-induced vapor generation. In a more-detailed comparison, the time for steam generation in the batch system was less than 5 s after illumination commenced. In our continuous system, the time for water flowing through the glass tube was ca. 1500 s. The energy density used in this work was still far lower than that for generating steam shown in the literature. It is well known that the required heat to break hydrogen bonds is 10~40 KJ mol^-1^, which is lower than that of 136 KJ mol^-1^ for evaporating water at room temperature. In this work, the water temperature did not significantly change when it passed through the glass tube with Au NPs under illumination. Therefore, the effect of hot electron transfer for breaking hydrogen bonds of bulk water was achieved under illumination with full-wavelength visible light and was further enhanced by wavelength-optimized resonant light.

Measured DNHBW values of 26.35%, 25.94%, 25.44%, 24.57%, and 22.20% were recorded for prepared HRHBW after its preparation for 0, 1, 2, 3, and 7 days (and stored in a light-free chamber at room temperature), respectively.

5. Evidence of more ‘free water’ by mixing with ethyl alcohol

Evidence of more ‘free water’ was further demonstrated by mixing with ethyl alcohol. DI water and HRHBW both at 10 wt% were individually dissolved in two samples of ethyl alcohol. The measured water contents using volumetric Karl Fischer titration (Metrohm 870 KF Titrino plus) were 11.2±0.18 and 10.2±0.14 wt% for DI water and HRHBW, respectively^44^. Because the HRHBW with weaker hydrogen bonds can form more hydrogen bonds with ethyl alcohol, the measured content of available water decreased by 8.9% compared to DI water.

6. Chemical activity of steam in the reduction preparation of Au NPs

As reported by Vohringer-Martinez et al.^45^, radical-molecule gas-phase reactions can be catalyzed by water molecules through their ability to form hydrogen bonds. Small gas-phase water clusters can bind to excess electrons through a double hydrogen-bond acceptor motif^46^. Therefore, the chemical activity of steam produced from HRHBW was evaluated on the reduction preparation of Au NPs from Au salts with the assistance of the weak reducing agent of Ch. As shown in Figure S2a, wine-red to purple aggregated Au NPs were found on the qualitative filter paper after experiments were performed with steam produced from HRHBW for 3 days. As discussed in the text, characteristics of HRHBW can last for at least 3 days. However, in the experiment performed using steam produced from DI water, no Au NPs were found on the qualitative filter paper. This indicates that the Au NPs had been successfully prepared with the assistance of the reducing agent of steam produced from HRHBW.

Figure S3 displays the HRXPS Au 4f_7/2-5/2_ core-level spectra of reduction preparations of Au NPs on qualitative filter paper via assistance of steam produced from HRHBW and from DI water. As shown in curve (a) based on steam from HRHBW, the doublet peaks were located at 84.0 and 87.7 eV, which can be assigned to Au(0) according to the XPS handbook and a report in the literature^47^. This confirms that the Au NPs from reduction preparation are in an elemental state. Comparing spectrum b (representing Au salts with positively charged Au-containing complexes after wetting with steam from DI water) with spectrum a (representing elemental Au(0) NPs), it was found that different Au components were shown in the region of higher binding energy for positively charged Au-containing complexes. The oxidized Au shown at 84.5 and 88.2 eV in spectrum b can respectively be assigned to monovalent Au(I) and trivalent Au(III)^48^. Moreover, the full widths at half maximum (FWHMs) of HRXPS Au 4f_7/2_ core-level spectra were 2.2 and 1.1 eV for experiments based on steam respectively produced from HRHBW and DI water. These results also confirmed their different oxidation states. It can be concluded, that steam from HRHBW indeed works for the reduction preparation of Au NPs from Au salts. However, this reduction did not occur when using steam from general bulk DI water, of when DI water was heated and maintained at 40 °C to producing additional steam. Because a similar experiment did not work without the assistance of the weak reducing agent of Ch, this chemical activity of reduction is weak. However, it opens a new green pathway for chemical reduction. We think more free water being available in the HRHBW with weaker hydrogen bonds is responsible for this novel activity.

7. Energy efficiency analysis of hydrogen evolution reaction

The energy efficiency, η, increased with HRHBW compared to bulk water in the hydrogen evolution reaction performed *in situ* of reducing hydrogen bonds under illumination with a green LED on a roughened Au substrate (main text, Figure 5c). This value was estimated from the ratio of the reduced cathodic overpotential (E_over, red_) required for hydrogen evolution reaction performed *in situ* of reducing hydrogen bonds under illumination with the green LED to the normally cathodic overpotential (E_over, nor_) required for hydrogen evolution reaction performed on DI water without illumination of light at a specific current yield, as defined here:

η = (E_over, red_) / E_over, nor_ × 100%. (3)

In this system, a reference electrode of Ag/AgCl in a saturated KCl solution was used. Thus the potential of this Ag/AgCl electrode relative to a standard hydrogen electrode (SHE) was 0.20 V vs SHE. Hydrogen evolution reaction was achieved in a solution containing 0.5 M H_2_SO_4_. Thus the equilibrium potential for hydrogen evolution reaction was 0.00 vs SHE. At a specific current yield of -10 mA, the E_over, nor_ was ca. -0.58 V vs SHE. The required cathodic overpotential was ca. -0.46 V vs SHE when the experiment was performed *in situ* of reducing hydrogen bonds under illumination with a green LED. Thus, the E_over, red_ was 0.12 V vs SHE, and the corresponding η was ca. 21%. Correspondingly, the increased energy efficiencies were 18%, 16%, and 14% at specific current yields of -20, -30, and -40 mA, respectively.

8. Influences of impurities on the treated water and on the corresponding hydrogen evolution

As shown in Figure 3a and 3b in the text, voltammetric data of CV (in 0.5 M H_2_SO_4_) and LSV (in H_2_SO_4_ between 0.05 and 0.5 M) for hydrogen evolution in DI water, RHBW, and HRHBW were demonstrated. Compared to the high concentrations of electrolytes of H_2_SO_4_ used in these voltammetric data for hydrogen evolution, the low concentration of slightly dissolved metals in RHBW, which was also slightly higher than that in DI water, was negligible for hydrogen evolution. Moreover, if the impurities were predominant in the efficient hydrogen evolution, the efficiencies in hydrogen evolution for RHBW and HRHBW in LSV data in Figure 3b should have been similar, because these two samples were prepared using the same supported Au NPs, but under different light sources. However, the observed efficiencies in hydrogen evolution greatly differed. This suggests that there was no influence from impurities on the corresponding efficiency of hydrogen evolution.

As shown in Figure 5c in the text for the other system, LSV data for hydrogen evolution in DI water containing 0.5 M H_2_SO_4_ with an electrochemically roughened Au electrode under illumination with different light sources (including light-free conditions) were demonstrated. In these experiments, breaking hydrogen bonds occurred at the illuminated Au electrode with Au NPs. The *in situ*-treated water was also contributive to the corresponding efficient hydrogen evolution. There was no issue regarding the influence of dissolved impurities on the improved hydrogen evolution because a blank experiment on the same Au electrode with Au NPs in a light-free condition was also performed.

To further address the issue of impurities, experiments of hydrogen evolution based on HRHBW at 0, 1, 3, 5, and 7 days after its preparation were performed. Because the prepared HRHBW decays with time, respective measured values of DNHBW were 26.35%, 25.94%, 25.44%, 24.57%, and 22.20% at 0, 1, 2, 3, and 7 days after its preparation. Therefore, if impurities were predominant in the efficient hydrogen evolution, the efficiencies of hydrogen evolution for experiments performed on different days after the preparation of HRHBW should have been similar, because these experiments were performed using the same original HRHBW with the same impurities.

9. Faraday efficiency in hydrogen production

The hydrogen productions based on systems of DI water and HRHBW in acidic solution (0.5 M H_2_SO_4_) at –0.6 V vs. Ag/AgCl were collected by water displacement and were quantified by gas chromatography (Model ACME 6000 series, Young Lin). The Faraday efficiencies in the electrochemical hydrogen evolution reactions to these systems were calculated by the following equation:


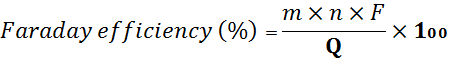


where *m* is the moles of produced H_2_; *n* is the number of electrons required for the production of H_2_ (n = 2); *F* is Faraday constant (96500 C/mol of electrons); and Q is the total charge in Coulombs passed across the Pt electrode during electrolysis. Therefore, the Faraday efficiency could be estimated. The calculated Faraday efficiencies are 84.0±5.4% and 86.2±3.5% for systems of DI water and HRHBW.

10. Highly efficient hydrogen evolution on a rough Au electrode using HRHBW

As shown in Figure 5c, the hydrogen evolution reaction (HER) was also evaluated on an Au NP-deposited Au electrode during illumination to reduce hydrogen bond preparations from DI water and *in situ* water electrolysis. Experimental results indicated that compared to a light-free condition (corresponding to bulk water), the efficiency of hydrogen evolution significantly increased by ca. 31% and 59% based on the *in situ* process of using fluorescent illumination and a green LED, respectively (in 0.5 M H_2_SO_4_ at -0.6 V)]. A similar experiment based on *in situ* treatment using the green LED was also performed, but DI water was replaced with HRHBW in that experiment. The corresponding results are shown in Figure S6. The efficiency of hydrogen evolution further obviously increased when DI water was replaced with HRHBW in the experiment. Compared to the light-free condition in DI water (corresponding to bulk water), the efficiency of hydrogen evolution in HRHBW significantly increased by ca. 84% based on *in situ* treatment using the green LED (in 0.5 M H_2_SO_4_ at -0.6 V). This novel increase in efficiency for hydrogen evolution indicates that reducing hydrogen bonds within water molecules is noticeable when splitting water.

**Table S1.** Ratios of five-Gaussian components of OH stretching Raman bands and the degree of non-hydrogen-bonded water (DNHBW) for various pure waters

| Sample | (1) 3018 cm^-1^ (%) | (2) 3223 cm^-1^ (%) | (3) 3393 cm^-1^ (%) | (4) 3506 cm^-1^ (%) | (5) 3624 cm^-1^ (%) | DNHBW (%) |
| --- | --- | --- | --- | --- | --- | --- |
| a | 4.99 | 38.85 | 34.71 | 15.7 | 5.76 | 21.46 |
| b | 3.76 | 38.27 | 33.74 | 16.95 | 7.29 | 24.24 |
| c | 5.23 | 39.72 | 33.54 | 15.71 | 5.8 | 21.50 |
| d | 2.78 | 37.18 | 33.81 | 18.97 | 7.26 | 26.23 |
| e | 5.26 | 40.17 | 33.33 | 15.40 | 5.83 | 21.24 |
| f | 1.19 | 35.30 | 33.20 | 20.36 | 9.95 | 30.31 |
| g | 4.59 | 37.96 | 33.1 | 17.57 | 6.78 | 24.36 |
| h | 3.70 | 38.24 | 33.82 | 17.84 | 6.29 | 24.23 |
| i | 4.49 | 35.72 | 34.84 | 20.11 | 4.83 | 24.94 |
| j | 4.86 | 39.44 | 32.83 | 16.77 | 6.09 | 22.86 |

Various samples of pure water: (a) DI water; (b) RHBW; (c) Blank; (d) HRHBW; (e) CPT water; (f) super HRHBW; (g) AgNT water; (h) PtNT water, (i) Au/AgNT water; (j) ORC-Au electrode illuminated water.

**Table S2.** Voltammetric data for the potentials of the first (E_p_^red(1)^) and second (E_p_^red(2)^) reduction peaks, and their potential differences (ΔE_p_) in various sample solutions, corresponding to Figure 2b

| Sample | E_p_^red(1)^ | E_p_^red(2)^ | ΔE_p_ |
| --- | --- | --- | --- |
| a | -1.31 V | -0.57 V | 0.74 V |
| b | -1.14 V | -0.50 V | 0.64 V |
| c | -1.11 V | -0.51 V | 0.60 V |
| d | -1.07 V | -0.5 V | 0.57 V |

Various sample solutions: (a) CH_3_CN without water; (b) CH_3_CN with DI water; (c) CH_3_CN with RHBW; (d) CH_3_CN with HRHBW.

**Table S3. Hydrogen evolution currents at -0.4 V vs Ag/AgCl in various waters with different concentrations of H_2_SO_4_, corresponding to Figure 3b**

| [H_2_SO_4_] | DI water | RHBW | HRHBW |
| --- | --- | --- | --- |
| 0.05 M | -0.944 mA | -1.19 mA | -1.13 mA |
| 0.10 M | -1.65 mA | -1.93 mA | -2.14 mA |
| 0.25 M | -3.19 mA | -3.58 mA | -4.24 mA |
| 0.50 M | -4.79 mA | -5.61 mA | -7.08 mA |

**Table S4** Performances of water splitting in DI water-based and HRHBW-based systems at various pH values

| pH value | Water type | Onset potential (V) | Overpotential (V) | Tafel slope  (mV/decade) |
| --- | --- | --- | --- | --- |
| -0.3 | DI | 0.217 | 0.27 (10 mA cm^-2^) | 0.043 |
|  | HRHBW | 0.205 | 0.24 (10 mA cm^-2^) | 0.041 |
| 7.0 | DI | 0.729 | 1.03 (0.5 mA cm^-2^) | 0.299 |
|  | HRHBW | 0.716 | 1.00 (0.5 mA cm^-2^) | 0.283 |
| 14.3 | DI | 0.951 | 1.08 (2.5 mA cm^-2^) | 0.075 |
|  | HRHBW | 0.942 | 1.07 (2.5 mA cm^-2^) | 0.071 |

**Table S5. Hydrogen evolution currents in DI water containing 0.5 M H_2_SO_4_ at an electrochemically roughened Au electrode under illumination with different light sources, corresponding to Figure 5c**

| Potential  vs Ag/AgCl | Light-free | Fluorescent lamp | Green LED |
| --- | --- | --- | --- |
| -0.4 V | -0.256 mA | -0.281 mA | -0.391 mA |
| -0.5 V | -0.813 mA | -1.17 mA | -1.64 mA |
| -0.6 V | -3.88 mA | -5.08 mA | -6.18 mA |
| -0.7 V | -10.8 mA | -12.5 mA | -13.7 mA |
| -0.8 V | -19.1 mA | -19.9 mA | -22.4 mA |

**
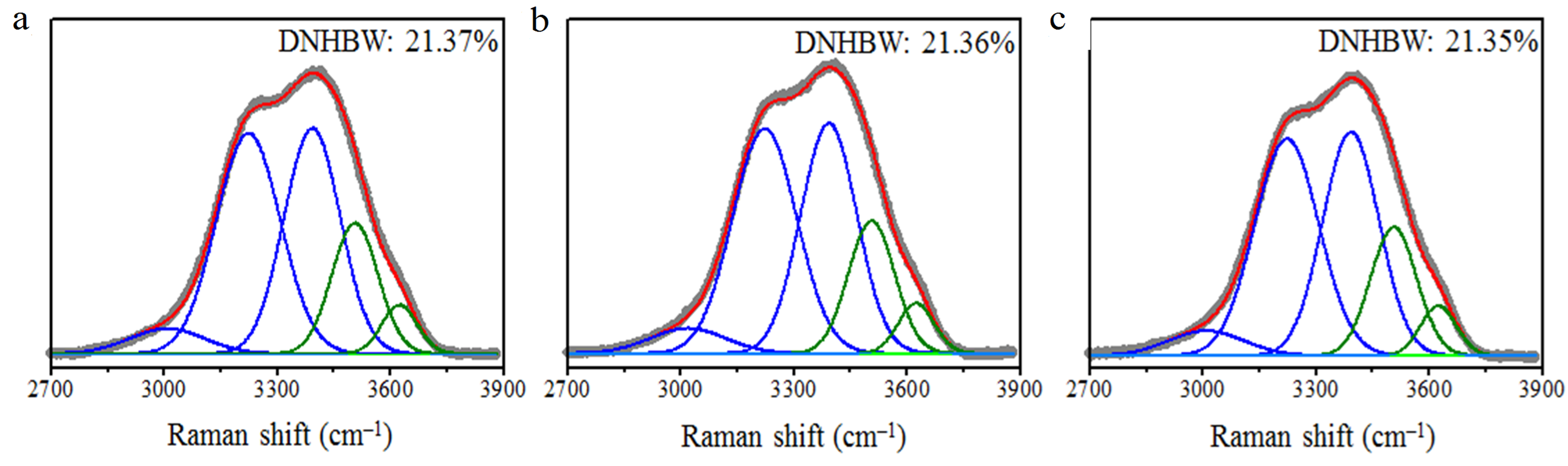
**

**Figure S1.** Raman spectra of OH stretching and the corresponding values of DNHBW of DI water, ceramic particles-treated water, and Au-coated ceramic particles-treated water, which are prepared at 50 ^o^C and measured at room temperature after cooling. (a) DI water, (b) Ceramic particles-treated water, and (c) Au-coated ceramic particles-treated water.

*
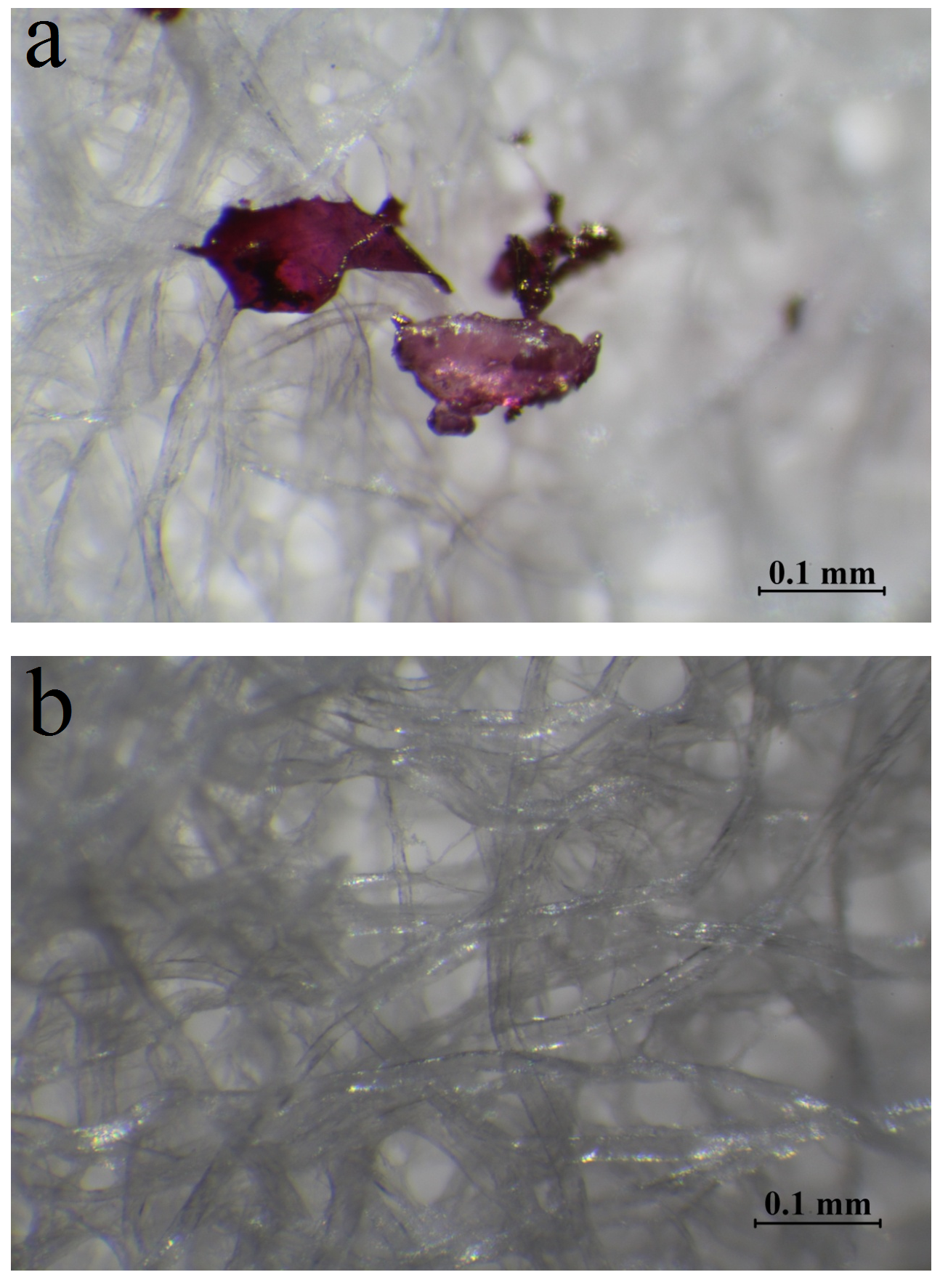
*

**Figure S2.** Optical microscopic images showing reduction preparation of Au NPs on qualitative filter paper via assistance of steam produced from various waters. (a) Steam from HRHBW. (b) Steam from DI water. Before the experiments, 10-mL stock solutions, which were prepared by dissolving fixed concentrations of Au salts and chitosan in DI water, were dropped onto the qualitative filter paper. Then the wetted papers were placed on the opening of two glass sample cells, which individually contained DI water or HRHBW, in ambient laboratory air for 3 days.

*
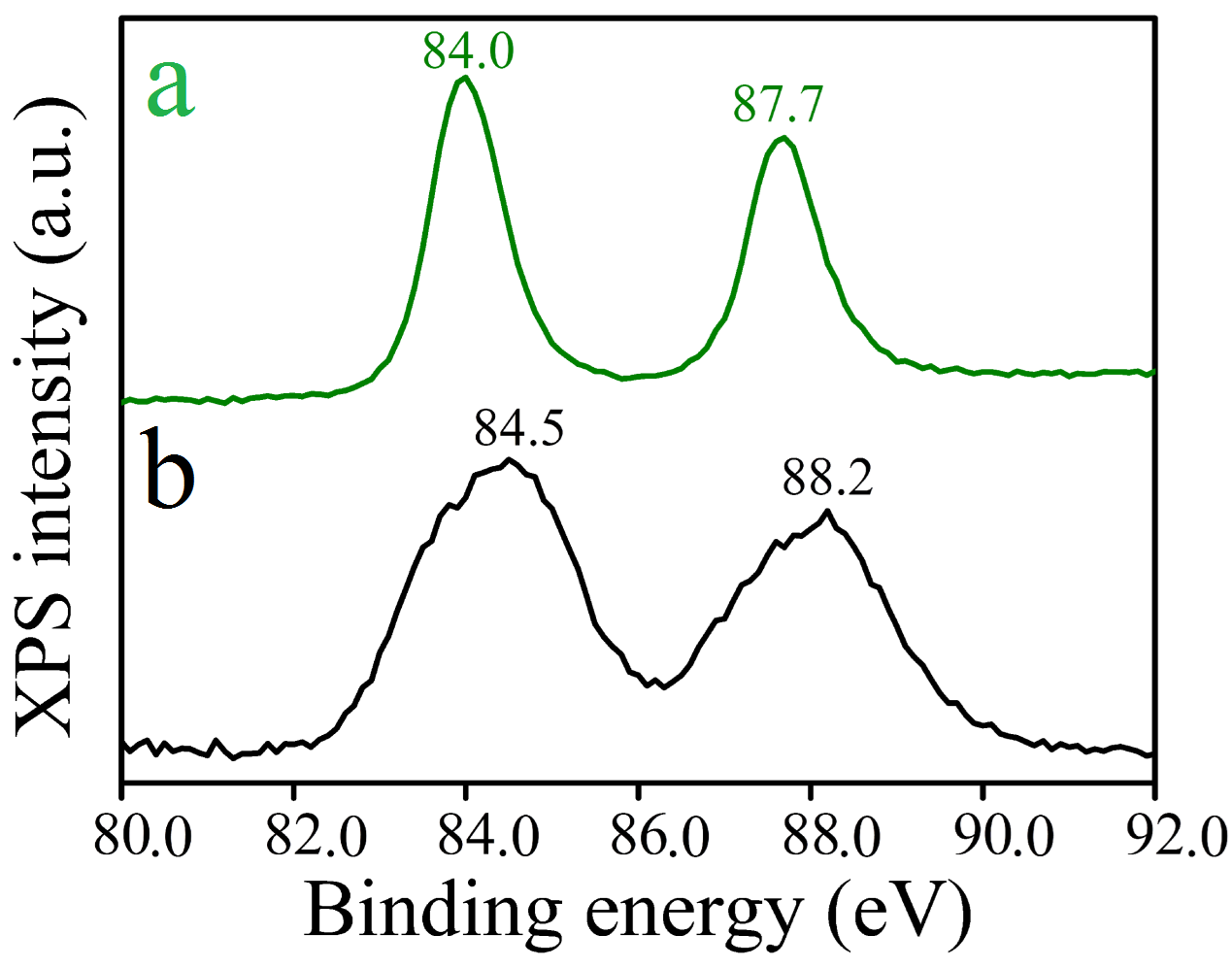
*

**Figure S3.** HRXPS Au 4f_7/2-5/2_ core-level spectra of reduction preparation of Au NPs on qualitative filter paper with the assistance of steam produced from various waters. (a) Steam from HRHBW. (b) Steam from DI water.

*
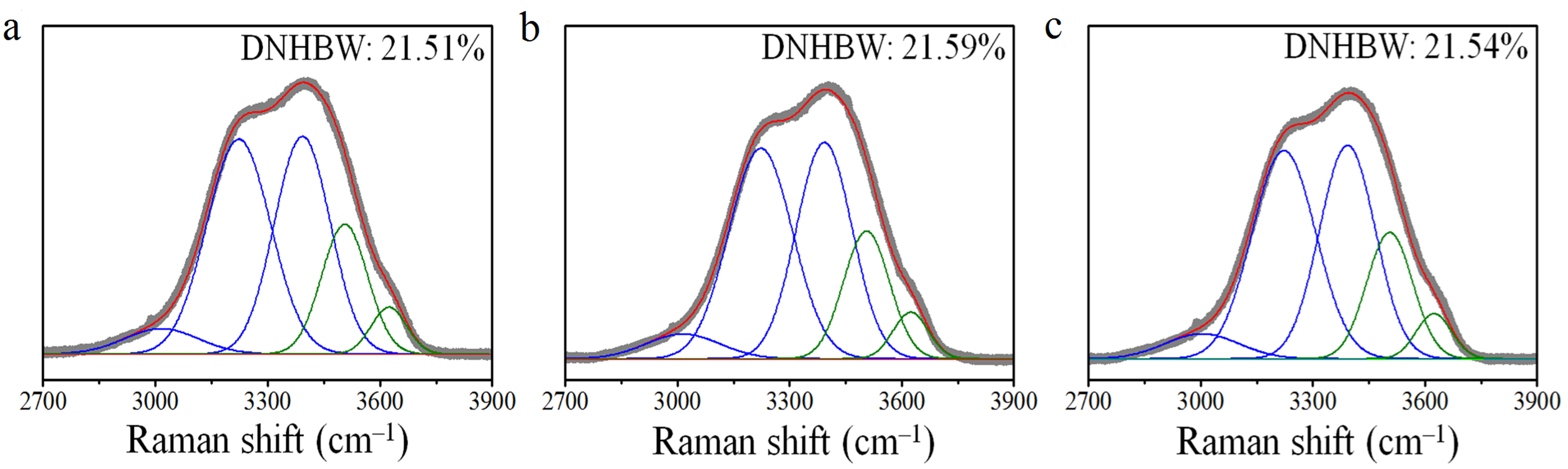
*

**Figure S4.** Raman spectra of OH stretching and the corresponding values of DNHBW of (a) DI water, (b) blank, and (c) blank sample (10 mL in a 20-mL glass sample cell) further illuminated with a green LED (λ_max_ 530 nm) for 1 h.

*
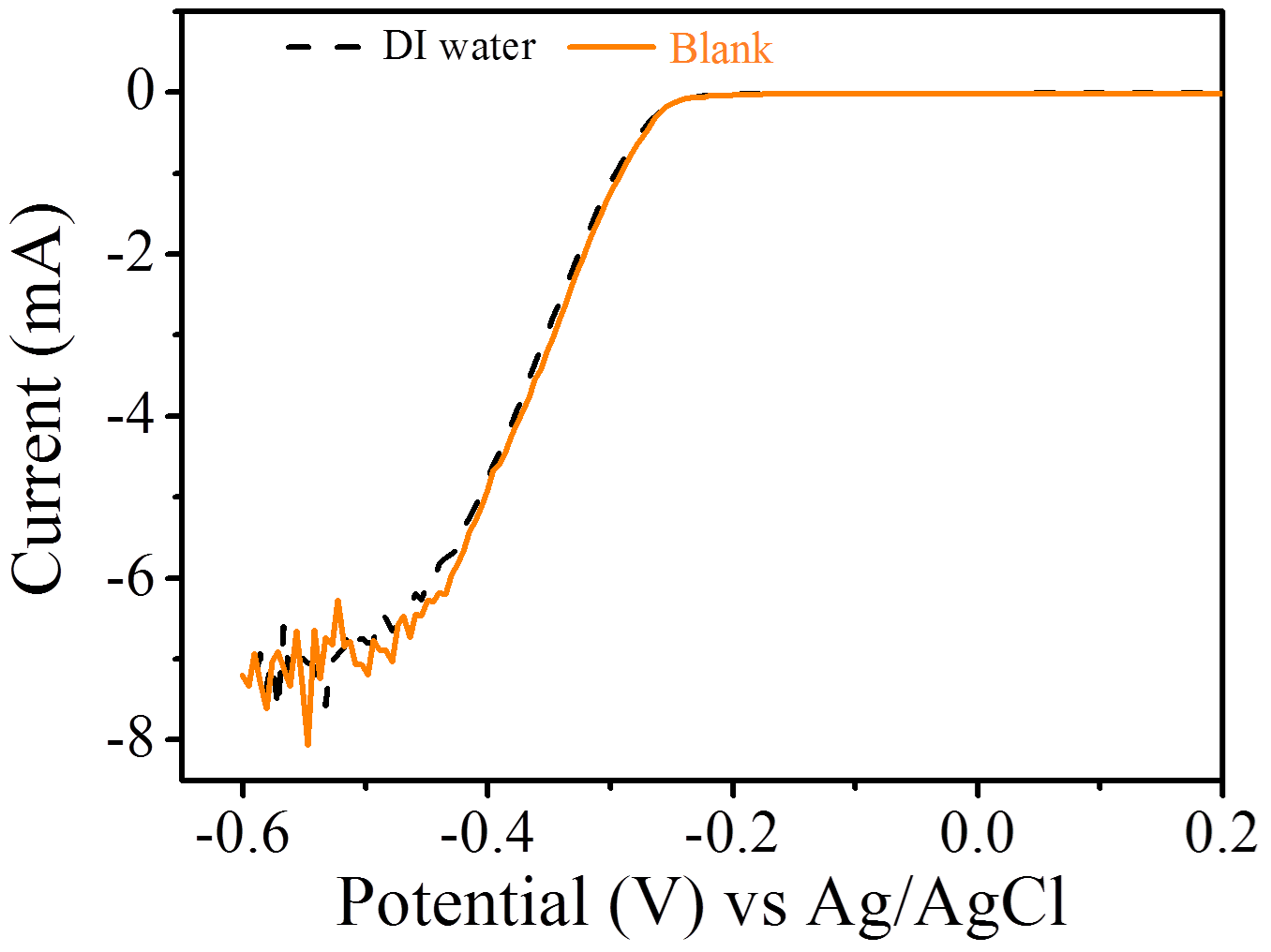
*

**Figure S5.** Linear scan voltammetric data for hydrogen evolution in various types of water with a 3-mm-diameter planar Pt electrode (in 0.5 M H_2_SO_4_ at a scan rate of 0.05 V s^-1^). Black and orange lines represent experiments performed in DI water and the blank, respectively.


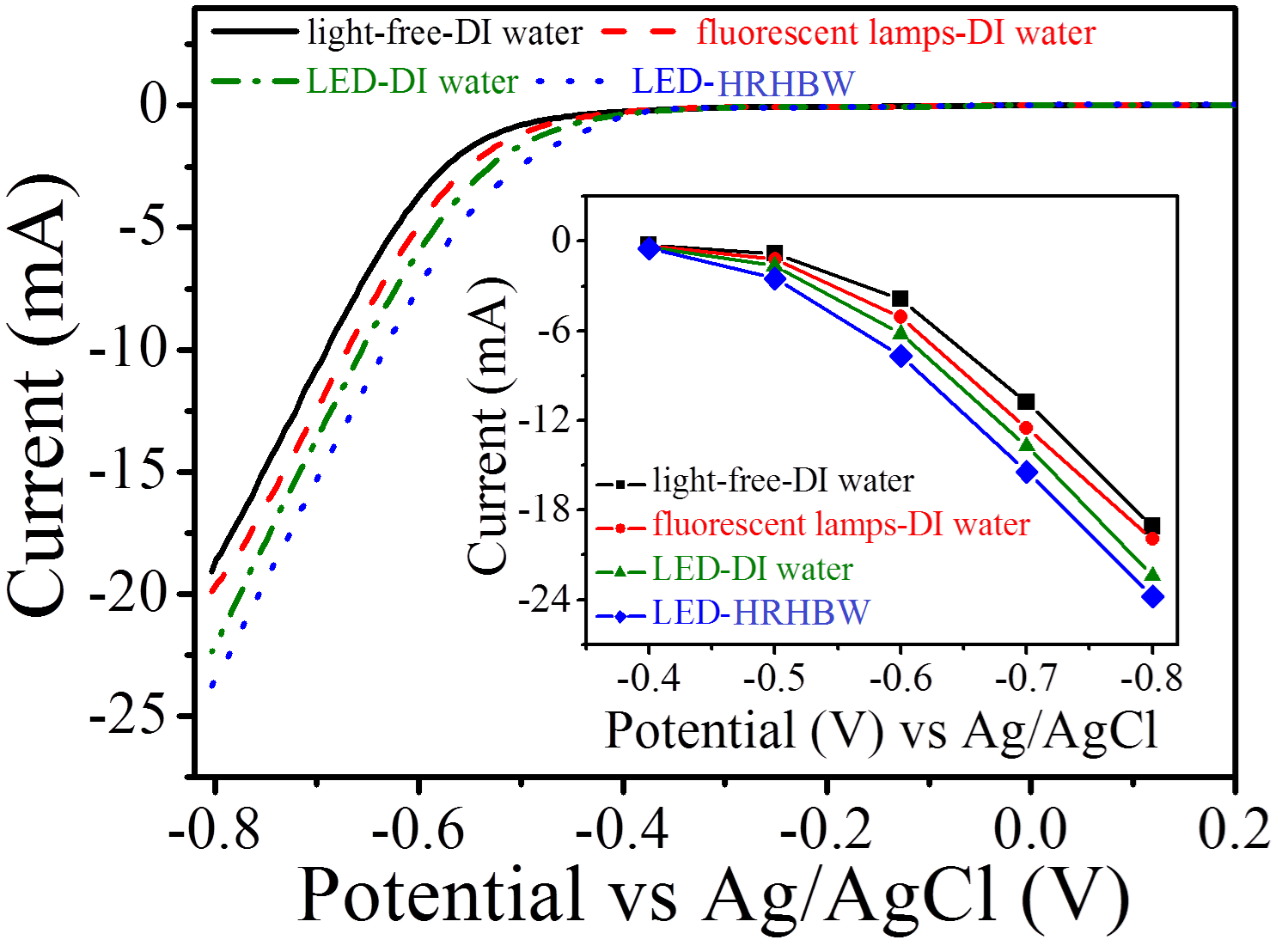


**Figure S6.** LSV of hydrogen evolution in DI water and HRHBW containing 0.5 M H_2_SO_4_ at an electrochemically roughened Au electrode under illumination with different light sources. The roughened Au electrode (0.238 cm^2^) was prepared with electrochemical oxidation-reduction cycles (ORCs) in 0.1 M KCl at 0.5 V s^-1^ for 25 scans, which was similar to that shown in SI for DNHBWs of differently treated waters; the insert shows HER currents at different cathodic potentials. Red and green lines respectively represent experiments performed in DI water under illumination with a fluorescent lamp and a green LED; the black line represents experiments performed in DI water with a light-free environment for reference. The blue line represents experiments performed in HRHBW under illumination with a green LED.


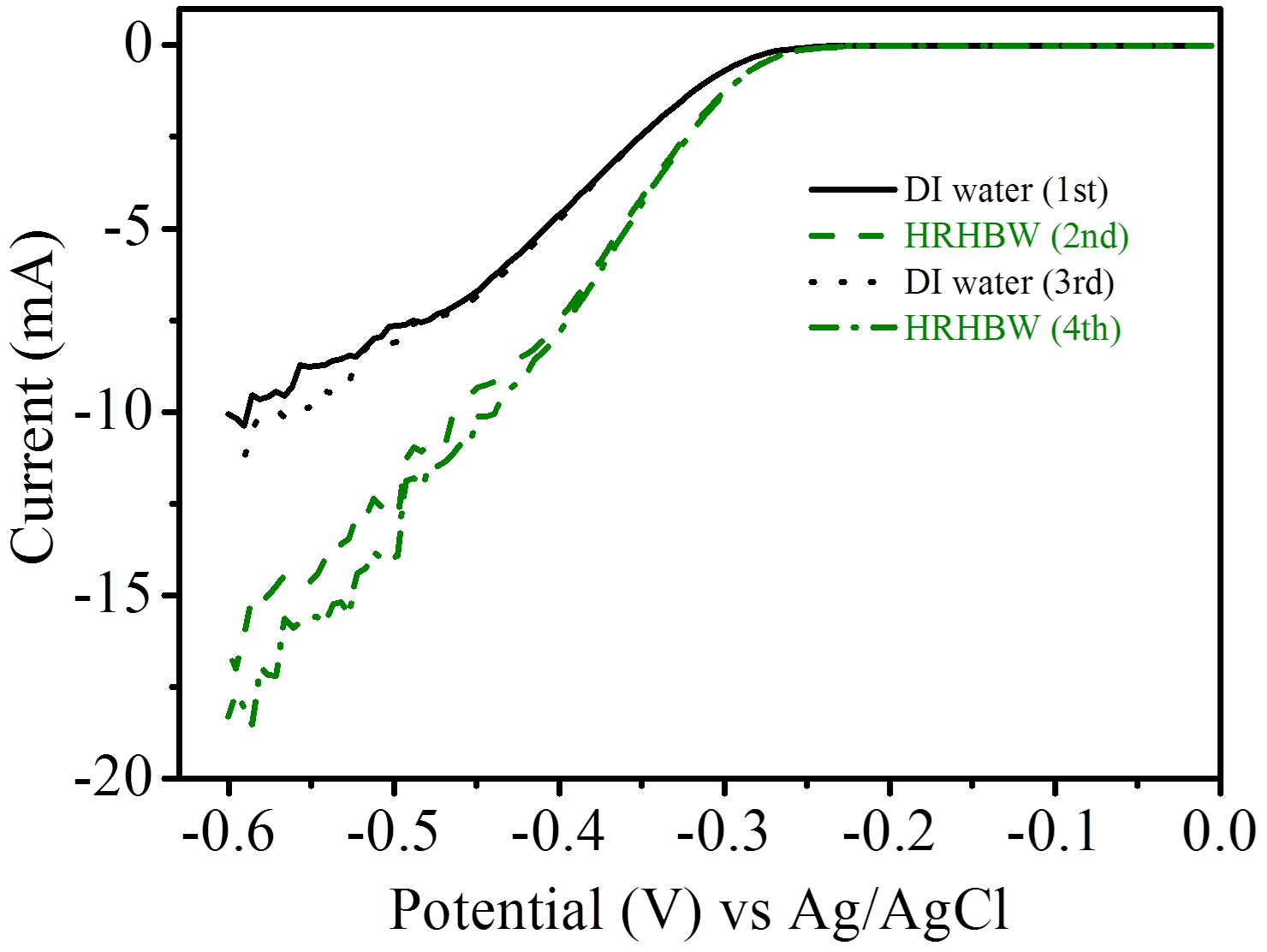


**Figure S7.** Linear sweep voltammograms (LSVs) for hydrogen evolution reaction (HER) recorded on a planar Pt electrode in 0.5 M H_2_SO_4_ at a scan rate of 0.05 V s^-1^ based on DI water and HRHBW. After the first HER an polished Pt electrode based on DI water, the used Pt electrode was just rinsed with DI water without further polishing treatment in sequent experiments based on DI water, HRHBW, DI water and HRHBW in sequence.

**References**

1. Wu, H.B., Xia, B. Y., Yu, L., Yu X. Y. & Lou, X. W. Porous ​molybdenum carbide nano-octahedrons synthesized via confined carburization in metal-organic frameworks for efficient ​hydrogen production. *Nat. Commun.* **6,** 6512 (2015).

2. Ji, L. *et al.* A silicon-based photocathode for water reduction with an epitaxial SrTiO_3_ protection layer and a nanostructured catalyst. *Nat. Nanotechnol.* **10,** 84–90 (2015).

3. Reece, S. Y. *et al.* Wireless solar water splitting using silicon-based semiconductors and earth-abundant catalysts. *Science* **334,** 645–648 (2011).

4. Vojvvodic, A. & Norskov, J. K. Optimizing perovskites for the water-splitting reaction. *Science* **334,** 1355–1356 (2011).

5. Andreiadis, E. S. *et al.* Molecular engineering of a cobalt-based electrocatalytic nano-material for H_2_ evolution under fully aqueous conditions. *Nat. Chem.* **5,** 48–53 (2013).

6. Yang, J., Wang, D., Han, H. & Li, C. Roles of cocatalysts in photocatalysis and photoelectrocatalysis. *Acc. Chem. Res.* **46,** 1900–1909 (2013).

7. Fujishima, A. & Honds, K. Electrochemical photolysis of water at a semiconductor electrode. *Nature* **238,** 37–38 (1972).

8. Yerga, R. M. N., Galvan, M. C. A., Del Valle, F., De la Mano, J. A. V. & Fierro, J. L. G. Water splitting on semiconductor catalysts under visible-light irradiation. *ChemSusChem* **2,** 471–485 (2009).

9. Armstrong, F. A. Copying biology’s ways with hydrogen. *Science* **339,** 658–659 (2013).

10. Khaselev, O. & Thrner, J. A. E. A monolithic photovoltaic-photoelectrochemical device for hydrogen production via water splitting. *Science* **280,** 425–427 (1998).

11. Symes, M. D. & Cronin, L. Decoupling hydrogen and oxygen evolution during water splitting using a proton-coupled-electron buffe. *Nat. Chem.* **5,** 403–409 (2013).

12. Li, R., Jiang, Z., Guan, Y., Yang, H. & Liu, B. Effects of metal ion on the water structure studied by the Raman O-H stretching spectrum. *J. Raman Spectrosc*. **40**, 1200-1204 (2009).

13. Carey, D. M. & Korenowski, G. M. Measurement of the Raman spectrum of liquid water. *J. Chem. Phys*. **108**, 2669-2775 (1998).

14. Tomlinson-Phillips, J., Davis, J. & Ben-Amotz, D. Structure and dynamics of water dangling OH bonds in hydrophobic hydration shells. Comparison of simulation and experiment. *J. Phys. Chem. A* **115,** 6177–6183 (2011).

15. David, J. G., Gierszal, K. P., Wang, P. & Ben-Amotz, D. Water structural transformation at molecular hydrophobic interfaces. *Nature* **491,** 582–585 (2012).

16. Turner, J. A. Sustainable hydrogen production. *Science* **305,** 972–974 (2004).

17. Li, J. F. *et al.* Shell-isolated nanoparticle-enhanced Raman spectroscopy. *Nature* **464,** 392–395 (2010).

18. A. M. Gobin, M. H. Lee, N. J. Halas, W. D. James, R. A. Drezek, J. L. West, *Nano Lett.* **7,** 1929–1934 (2007).

19. Chen, H. C. *et al.* Active and stable liquid water innovatively prepared using resonantly illuminated gold nanoparticles. *ACS Nano* **8,** 2704–2713 (2014).

20. Tessensohn, M. E., Hirao, H. & Webster, R. D. Electrochemical properties of phenols and quinones in organic solvents are strongly influenced by hydrogen-bonding with water. *J. Phys. Chem.* *C* **117,** 1081–1090 (2013).

21. Margolis, S. A. Source of the difference between the measurement of water in hydrocarbons as determined by the volumetric and coulometric Karl Fischer methods. *Anal. Chem.* **71,** 1728–1732 (1999).

22. Remes, A. *et al.* Electrochemical determination of pentachlorophenol in water on a multi-wall carbon nanotubes-epoxy composite electrode. *Sensors* **12,** 7033–7046 (2012).

23. Subbaraman, R. *et al.* Enhancing hydrogen evolution activity in water splitting by tailoring Li^+^-Ni(OH) 2-Pt interfaces. *Science* **334,** 1256–1260 (2011).

24. Huang, M. *et al.* Designed nanostructured Pt film for electrocatalytic activities by underpotential deposition combined chemical replacement techniques. *J. Phys. Chem. B* **109,** 15264–15271 (2005).

25. Hou, Y. D. *et al.* Layered nanojunctions for hydrogen-evolution catalysis. *Angew. Chem., Int. Ed.* **52,** 3621−3625 (2013).

26. Wang, Y., Wang, X. C. & Antonietti, M. Polymeric graphitic carbon nitride as a heterogeneous organocatalyst: from photochemistry to multipurpose catalysis to sustainable chemistry. *Angew. Chem., Int. Ed.* **51,** 68−69 (2012).

27. Zhang, S. *et al.* In situ synthesis of water-soluble magnetic graphitic carbon nitride photocatalyst and its synergistic catalytic performance. *ACS Appl. Mater. Interfaces.* **5,** 12735−12743 (2013).

28. Voiry, D. *et al.* Enhanced catalytic activity in strained chemically exfoliated WS_2_ nanosheets for hydrogen evolution. *Nat. Mater.* **12,** 850–855 (2013).

29. Esposito, D. V., Hunt, S. T., Kimmel, Y. C. & Chen, J. G. A new class of electrocatalysts for hydrogen production from water electrolysis: metal monolayers supported on low-cost transition metal carbides. *J. Am. Chem. Soc.* **134,** 3025–3033 (2012).

30. Zhao, Y., Kamiya, K., Hashimoto, K. & Nakanishi, S. Hydrogen evolution by tungsten carbonitride nanoelectrocatalysts synthesized by the formation of a tungsten acid/polymer hybrid in situ. *Angew. Chem. Int. Ed.* **52,** 13638–13641 (2013).

31. Zou, X. *et al.* Cobalt-embedded nitrogen-rich carbon nanotubes efficiently catalyze hydrogen evolution reaction at all pH values. *Angew. Chem. Int. Ed.* **53,** 4372–4376 (2014).

32. Kiani, A. & Hatami, S. Fabrication of platinum coated nanoporous gold film electrode: a nanostructured ultra low-platinum loading electrocatalyst for hydrogen evolution reaction. *Int. J. Hydrogen Energy* **35,** 5202–5209 (2010).

33. Vila`, N. *et al.* Metallic and bimetallic Cu/Pt species supported on carbon surfaces by means of substituted phenyl groups. *J. Electroanal. Chem.* **609,** 85–93 (2007).

34. Sun, L. S., Ca, D. V. & Cox, J. A. Electrocatalysis of the hydrogen evolution reaction by nanocomposites of poly(amidoamine)-encapsulated platinum nanoparticles and phosphotungstic acid. *J. Solid State Electrochem.* **9,** 816–822 (2005).

35. Ojani, R., Raoof, J. B. & Hasheminejad, E. One-step electroless deposition of Pd/Pt bimetallic microstructures by galvanic replacement on copper substrate and investigation of its performance for the hydrogen evolution reaction. *Int. J. Hydrogen Energy* **38,** 92–99 (2013).

36. Trueba, M., Trasatti, S. P. & Trasatti, S. Electrocatalytic activity for hydrogen evolution of polypyrrole films modified with noble metal particles. Mater. Chem. Phys. **98,** 165–171 (2006).

37. Lee, H. M. & Kim, K. S. Dynamics and structural changes of small water clusters upon ionization. *J. Comput. Chem.* **34,** 1589–1597 (2013).

38. Yu, C. C., Liu, Y. C., Yang, K. H. & Tsai, H. Y. Simple method to prepare size-controllable gold nanoparticles in solutions and their applications on surface-enhanced Raman scattering. *J. Raman Spectrosc.* **42,** 621–625 (2011).

39. Liu, Y. C., Yu, C. C. & Yang, K. H. Active catalysts of electrochemically prepared gold nanoparticles for the decomposition of aldehyde in alcohol solutions. *Electrochem. Commun*. **8,** 1163–1167 (2006).

40. Senior, W. A. & Thompson, W. K. Assignment of the infra-red and Raman bands of liquid water. *Nature* **205,** 170–170 (1965).

41. Neumann, O. *et al.* Solar vapor generation enabled by nanoparticles. *ACS Nano* **7,** 42–49 (2013).

42. Fang, Z. *et al.* Evolution of light-induced vapour generation at a liquid-immersed metallic nanoparticle. *Nano Lett.* **13,** 1736–1742 (2013).

43. Polman, A. Solar steam nanobubbles. *ACS Nano* **7,** 15–18 (2013).

44. Margolis, S. A. Source of the difference between the measurement of water in hydrocarbons as determined by the volumetric and coulometric Karl Fischer methods. *Anal. Chem.* **71,** 1728–1732 (1999).

45. Vöhringer-Martinez, E. *et al.* Water catalysis of a radical-molecule gas-phase reaction. *Science* **315,** 497–501 (2007).

46. Hammer, N. I. *et al.* How do small water clusters bind an excess electron? *Science* **306,** 675–679 (2004).

47. Henry, M .C., Hsueh, C. C., Timko, B. P. & Freund, M. S. Reaction of pyrrole and chlorauric acid a new route to composite colloids. *J. Electrochem. Soc.* **148,** D155–162 (2001).

48. Suzer, S., Ertas, N., Kumser, S. & Ataman, O. Y. X-ray photoelectron spectroscopic characterization of Au collected with atom trapping on silica for atomic absorption spectrometry. *Appl. Spectrosc.* **51,** 1537–1539 (1997).
